# Impact of constant low gas pressure on cardiopulmonary parameters and surgical outcomes in extraperitoneal total nerve-sparing robot-assisted radical prostatectomy

**DOI:** 10.3389/fsurg.2025.1702676

**Published:** 2026-02-09

**Authors:** Giovanni Cochetti, Alessio Paladini, Andrea Vitale, Matteo Mearini, Rachele Simonte, Francesco Oliva, Davide Valeri, Edoardo De Robertis, Ettore Mearini

**Affiliations:** 1 Urologic Clinic, Department of Medicine and Surgery, University of Perugia, Perugia, Italy; 2 Anesthesia, Analgesia, and Intensive Therapy, Department of Surgical and Biomedical Sciences, University of Perugia, Perugia, Italy

**Keywords:** extraperitoneal, general anesthesia, low insufflation pressure, minimally invasive surgical procedures, prostatectomy, RARP, robot-assisted

## Abstract

Traditionally, in extraperitoneal robot-assisted radical prostatectomy (EP-RARP), a pneumo-Retzius is obtained by using a CO_2_ insufflation pressure of 12–15 mmHg. However, EP surgery is associated with an increase in CO_2_ absorption and consequent hypercapnia and acidosis. This study aimed to compare the effect of low CO_2_ pressure (8 mmHg) with the conventional gas pressure in EP-RARP. We enrolled patients with low-risk prostate cancer who had undergone total nerve-sparing RARP using our PERUSIA (Posterior, Extraperitoneal, Robotic, Under Santorini, Intrafascial, Anterograde) technique. The exclusion criteria were the presence of chronic lung disease, a positive biopsy core from the anterior zone, or a shift to a transperitoneal approach. Cardiopulmonary parameters were measured at the induction of anesthesia (T0); at 5 (T1) and 60 (T2) minutes after starting CO_₂_ insufflation; and immediately after dorsal venous complex dissection before urethro-vesical anastomosis (T3). Data from 120 consecutive patients were retrospectively analyzed from a prospectively maintained database. Patients were divided into two groups based on the CO_2_ insufflation pressure (8 vs. 12 mmHg). No significant differences were detected in mean operative time, time required for trocar positioning, mean estimated blood loss, or complications between the two groups. The only significant difference was in the partial pressure of carbon dioxide, which was higher at T3 in Group 2 (*p*=0.005), with a consequent reduction in arterial pH. However, no significant difference (*p* = 0.44) was found regarding acidosis between the two groups at all timepoints. RARP has become a standard procedure in urological surgery for the treatment of localized prostate cancer. However, the CO_2_ insufflation required to create a surgical workspace may lead to cardiopulmonary complications, especially in patients with pre-existing respiratory conditions. This study compared the effects of a lower insufflation pressure (8 mmHg) vs. the standard pressure (12 mmHg) during EP-RARP. The findings suggest that using a low and constant pressure can reduce CO_2_ absorption into the bloodstream without increasing intraoperative or postoperative complications. This approach may expand eligibility for EP-RARP to include patients with chronic pulmonary diseases by enhancing the safety and tolerability of the procedure.

## 1 Introduction

Prostate cancer (PCa) remains a major public health concern among men, mainly due to increased life expectancy and population aging (1, 2). Robot-assisted surgery is now a standard approach for therapeutic procedures in urology (3, 4). The robot-assisted approach combines the surgical steps from open surgery with the benefits of a minimally invasive technique, overcoming the limitations of laparoscopy (5, 6). Robot-assisted radical prostatectomy (RARP) can be safely performed using either a trans- (TP) or extraperitoneal (EP) approach (7). The majority of surgeons prefer the transperitoneal (TP) approach due to the larger working space and the possibility of conducting a more extensive lymph node dissection. In contrast, the EP approach seems to guarantee a shorter operative time and lower complication rate, is closer to open surgical principles, and reduces the risk of postoperative ileus and intraoperative bowel injuries because the peritoneal cavity is not violated (8). However, the higher chance of anesthesiologic complications during RARP has been historically regarded as a contraindication for patients with chronic pulmonary diseases, severe heart conditions, hemodynamic instability, and increased intracranial pressure. Despite several studies comparing the oncological and functional results of EP- and TP-RARP, few have focused on the cardiopulmonary effects of the two techniques, especially in patients with impaired respiratory function (9, 10).

Hypercapnia during laparoscopic procedures can result from the absorption of CO_2_ into the bloodstream from open vessels or from an increase in dead space ventilation. Recent studies have shown that EP surgery is associated with higher CO_2_ absorption than TP, with consequent hypercapnia and risk of acidosis (3, 7, 9). We hypothesized that performing a dorsal venous complex-sparing (DVC-sparing) technique during an extraperitoneal robot-assisted radical prostatectomy (EP-RARP) with a gas pressure lower than the venous central pressure (VCP), approximately 10 mmHg, could reduce gas absorption into the bloodstream during venous exposition.

The primary aim of our study was to compare the effects on the cardiopulmonary system and peri-operative outcomes of a constant low-pressure (8 mmHg) CO_2_ pneumo-Retzius, generated and steadily maintained using the AirSeal® System (SurgiQuest, Milford, Connecticut, USA) (11), with the conventional pressure (12 mmHg) during extraperitoneal DVC-sparing RARP.

## 2 Materials and methods

The present study was conducted in a high-volume tertiary institute. After institutional and ethical review board approval, from January 2020 to July 2023, all consecutive patients with low-risk clinically localized PCa who were eligible for the total nerve-sparing PERUSIA (Posterior, Extraperitoneal, Robotic, Under Santorini, Intrafascial, Anterograde) technique were enrolled retrospectively and analyzed (12). The patients were grouped according to the CO_₂_ pressure used during their surgery, i.e., 8 mmHg (Group 1) or 12 mmHg (Group 2). The choice of insufflation pressure was due to a progressive change in institutional practice and was not based on the patient’s clinical or anesthesiologic characteristics. Each procedure was performed by a single skilled surgeon (EM) and a dedicated anesthesiology team, using a robot-assisted approach to reduce the impact of the treatment on the results. The treating physician was not blinded to the insufflation pressure. All the participants provided written informed consent prior to enrollment. The PCa risk category of the patients was classified according to the European Association of Urology (EAU) guidelines (13). The American Society of Anesthesiology (ASA) score and Charlson Comorbidity Index were used to classify their performance status and comorbidity, respectively (14, 15). Perioperative complications were reported according to the Clavien–Dindo classification (16). The inclusion criteria were as follows: low-risk organ-confined PCa, life expectancy >10 years, and a score of >17 on the five-question International Index of Erectile Function questionnaire (IIEF-5) score, indicating potency. Patients with high- or intermediate-risk PCa, those with positive biopsy cores from the anterior zone who were consequently not eligible for DVC preservation, those with severe chronic pulmonary disease, and those who required a shift to the TP approach were excluded.

Preoperative data were collected for all the patients. Hemodynamic, respiratory, and blood acid–base parameters were measured at T0 (after induction), T1 (5 min after insufflation), T2 (60 min after insufflation), and T3 (immediately after DVC dissection and before urethro-vesical anastomosis). The measurement at T3 was always performed immediately after the end of DVC dissection and before performing the urethro-vesical anastomosis. Although this timepoint was procedure-based rather than strictly time-based, the use of a standardized surgical technique performed by a single surgeon ensured minimal inter-patient variability. Continuous variables are presented as medians and interquartile ranges (IQRs) in Table 1. Intraoperative parameters are presented as means (±SDs) and the differences between the two groups are shown in Table 2.

**Table 1:** Patients’ preoperative parameters.

Variable	Group 1 (8 mmHg)	Group 2 (12 mmHg)	*P*-value
Age (years), median (IQR)	62 (51–68)	60 (51–68)	0.43
ASA score, median (IQR)	2 (1–2)	2 (1–2)	0.82
aaCCI score, median (IQR)	3 (0–6)	3 (0–6)	0.77
Clinical stage			
T1c, *n* (%)	32 (53.3)	37 (63.8)	0.26
T2a, *n* (%)	26 (43.3)	25 (36.2)	0.85
Variable			
Preoperative prostate-specific antigen (ng/mL), median (IQR)	6.0 (2.2–10)	6.2 (2.5–9.8)	0.68
Prostate volume (g), median (IQR)	44.5 (35.9–54.1)	45.5 (36–55)	0.91
IIEF-5 (preoperative), median (IQR)	21 (16–22)	23 (17–24)	0.48
International Prostate Symptom Score (preoperative), median (IQR)	9.5 (5–15)	10.5 (6–15)	0.60
BMI (kg/m^2^), median (IQR)	23 (21–27)	23 (21–28)	0.74

**Table 2:** Intra-operative parameters analysed at the three times.

Parameters	Group 1 (60) Mean (SD)				Group 2 (58) Mean (SD)						
	T0	T1	T2	T3	T0	T1	*p*	T2	*p*	T3	*p*
pH	7.404 (0.02)	7.375 (0.03)	7.385 (0.05)	7.355 0.05)	7.405 (0.02)	7.404 (0.04)	0.831	7.361 (0.05)	0.188	7.338 (0.05)	0.189
Arterial pO_2_ (mmHg)	137 (12.32)	207.75 (99.41)	116.9 (27.92)	104.81	141 (11.4)	180.01 (64.50)	0.408	113.82 (36.72)	0.420	131.81 (39.4)	0.880
Arterial pCO_2_ (mmHg)	33.6 (1.2)	39.20 (4.16)	39.06 (7.29)	36.03 (6.64)	33.7 (1.1)	38.36 (6.12)	0.916	39.96 (3.08)	0.130	43.2 (6.9)	0.005
End-tidal CO_2_ (mL)	26 (1.1)	31 (2.32)	28 (3.22)	31.3 (4.60)	25 (1.2)	34 (1.87)	0.583	31.5 (2.20)	0.703	31 (3.03)	0.650
Fraction of inspired oxygen (*Fi*O_2_)	21.23 (4.3)	32.14 (8.6)	33.89 (7.22)	33.33 (6.58)	21.34 (3.7)	29.792 (7.76)	0.178	40.667 (3.27)	0.090	30.792 (5.70)	0.146
Respiratory rate	12 (1.01)	12 (1.16)	12 (1.14)	12 (1.18)	12 (1.03)	12 (1.17)	0.983	12 (1.16)	0.980	13 (1.22)	0.984
Hb	13.7 (0.3)	13.6 (1.2)	13.4 (1.40)	12.5 (1.59)	13.3 (0.2)	13.2 (1.02)	0.984	12.6 (0.95)	0.265	12.6 (1.35)	0.493
Hct	40.53 (2.3)	40.51 (4.08)	37.71 (4.34)	41.61 (4.64)	41.21 (2.5)	41.11 (3.8)	0.830	38.11 (3.87)	0.341	38.21 (11.7)	0.282

An EP space was digitally created. We placed an optical trocar 1 cm below the navel and two robotic trocars were placed 7 cm laterally, avoiding the rectus abdominis muscle. The remaining robotic trocars were placed under laparoscopic vision 2 cm above and medial to the anterior-superior iliac spine, with one on the left and one on the right, for the AirSeal® bed assistant. The patient was then placed in the 18° Trendelenburg position (17). After the induction of a pneumo-Retzius and docking of the robot (Da Vinci Xi®, Intuitive Surgical, Sunnyvale, CA, USA), we performed a total nerve-sparing EP-RARP using the PERUSIA technique, which is characterized by DVC preservation. Since we only included patients with low-risk PCa according to the EAU risk group, a lymphadenectomy was never performed. In these patients, the risk of lymph node involvement never exceeded 2%, as indicated by the use of Memorial Sloan Kettering Cancer Centre PCa nomograms (18, 19).

Non-invasive [electrocardiogram, pulse oximetry, neuromuscular monitoring, train of four, and body temperature] and invasive monitoring (intra-arterial blood pressure and blood gas analysis) were performed. The patients underwent general anesthesia induction with fentanyl 2 µg/kg, propofol 2 mg/kg, and rocuronium 0,6 mg/kg; after endotracheal intubation, anesthesia was maintained with sevoflurane and additional boluses of rocuronium to maintain a deep neuromuscular blockade. The patients were ventilated using pressure-controlled ventilation with 40% oxygen, a positive end-expiratory pressure of 3–4 mmHg in order to maintain a tidal volume of 6–8 mL/Kg, and an end-tidal carbon dioxide pressure of 30–35 mmHg, which was carefully monitored with blood gas reports in parallel to check its suitability. In our study, the end-tidal CO_2_ value was used to estimate the patients’ pulmonary perfusion, with the normal gradient considered to be constant at approximately 5 mmHg in an anesthetized man without any pulmonary disease (20). No patients developed clinically significant acidosis that required a corrective anesthesiologic intervention, and the ventilation parameters were managed according to a standardized protocol in both groups.

Blood gas values at the three different timepoints were compared using repeated measures ANOVA with the Bonferroni correction for pairwise comparisons. Data are presented as mean ± SD or median (IQR), as appropriate. Continuous variables were compared using Student's *t*-test or the Mann–Whitney *U*-test, and categorical variables with *χ*^2^ or Fisher's exact test. All *p*-values less than 0.05 were considered statistically significant. All the analyses were performed using SPSS® (IBM, NY, USA).

## 3 Results

A total of 120 patients who underwent total nerve-sparing EP-RARP using the PERUSIA technique were included in this study. The patients were divided into two groups according to the gas insufflation pressure used in their surgery, i.e., the low-gas-pressure group, with 60 patients (Group 1), and the standard-gas-pressure group, with 60 patients (Group 2). The use of the age-adjusted Charlson Comorbidity Index (aaCCI) and ASA score indicates that the patients had few comorbidities. Table 1 presents a summary of the preoperative parameters.

### 3.1 Perioperative surgical outcomes

No significant differences were found between the groups for operative time (137.2 min vs. 137 min, *p* = 0.97), trocar positioning time (15 min vs. 15.3 min, *p* = 0.82), and estimated blood loss (210 mL vs. 230 mL, *p* = 0.44). Heart rate variations were also not significant (*p* = 0.61). Hemoglobin (Hb) and hematocrit (Hct) levels were recorded during blood gas testing at each timepoint and no differences were detected in the mean value at the three analyzed timepoints between the two groups. The recorded Grade 1 complications included transient fever and mild subcutaneous emphysema; Grade 2 complications were urinary tract infection and blood transfusion. There were no Grade ≥3 events, results are reported in Table 3. There was no significant difference in complication rate between the groups (*p* = 0.78), and no major complications were recorded in either group. Two patients in Group 2 were excluded from the analysis because of the occurrence of small lacerations in the peritoneum, which required a shift to the TP approach in order to reduce confounding.

**Table 3:** Complication rate and type recorded in the two groups.

	Group 1 (60) (%)	Group 2 (58) (%)	*P*-value
Overall	4 (6.6)	3 (5.2)	0.72
Grade I	2 (3.3)	1 (1.8)	0.56
Grade II	2 (3.3)	2 (3.4)	1
Grade III	0	0	–
Grades IV and V	0	0	–

### 3.2 Intraoperative anesthesiologic outcomes

The analysis of the intraoperative cardiopulmonary parameters showed that the partial pressure of carbon dioxide (PaCO_2_) was only significantly higher at T3 (*p* = 0.005) in Group 2, with a consequent reduction in arterial pH. However, no significant difference (*p* = 0.44) was found in acidosis between the two groups at all timepoints. No differences in the other parameters were recorded between the two groups (Table 2).

## 4 Discussion

Currently, RARP is an established treatment for localized PCa, and EP-RARP and TP-RARP have been demonstrated to have equivalent efficacy (8, 21). During laparoendoscopy, CO_2_ insufflation into the peritoneal and extraperitoneal cavities produces a wide range of hemodynamic and cardiopulmonary changes that must be monitored and managed in order to prevent complications. Although many studies have documented these changes, only a few reports identified different anesthesiologic effects between the two approaches (7, 9, 22). The impossibility of maintaining constant intra-cavity pressure throughout the operating phases is a major limitation of many previous reports that evaluated intraoperative parameters (7, 9, 23). Regardless of the approach, the continuous presence of positive and negative pressure peaks could have negative effects on cardiopulmonary function if alveolar ventilation is not adequately set (24). Furthermore, little is known about the mechanism of gas absorption during endoscopic procedures.

We compared intraoperative cardiopulmonary parameters during standard-pressure EP-RARP (12 mmHg) with those during constant low-pressure EP-RARP (8 mmHg). No cases of acidosis were recorded, perhaps because these patients were able to easily adapt to the increase in end-tidal CO_2_ volume.

A previous experimental study hypothesized that an increase in insufflation pressure from 0 to 10 mmHg would expose a larger part of the peritoneum to the gas and, therefore, result in an increased diffusion area. However, the study found that a further increase in intra-cavity pressure did not result in a larger diffusion area but instead caused a plateau in CO_2_ insufflation (25). It is unclear whether the same mechanisms influence CO_2_ diffusion during EP surgery. Kanwer et al. compared the hemodynamic effects of using a low CO_2_ pressure (10 mmHg) during TP cholecystectomy with the use of a standard pressure pneumoperitoneum (14 mmHg), concluding that there were no statistically significant changes in blood pressure or heart rate (26). Glascock et al. suggested that direct intravascular uptake of CO_2_ could also be a consequence of the disruption of microvascular and lymphatic channels, especially in the case of bleeding during RARP. Moreover, the dissection along fascial tissue may also support the passage of CO_2_ along these planes, facilitating gas absorption into pre-peritoneal capillary beds (23). This could particularly be true when using a trocar-mounted balloon dilator device or a robotic camera through a blunt Hasson trocar in order to develop the extraperitoneal space. For this reason, we prefer to develop EP space using a laparoscopic approach in order to reduce the risk of microscopic defects in the peritoneal membrane. This reduces the rate of subcutaneous emphysema, which is a further non-negligible mechanism of higher CO_2_ absorption during EP surgery, considering its high prevalence during the development of a smaller working space (27).

Another proposed mechanism to explain enhanced CO_2_ absorption is the disruption of the lymph vascular channels during the tearing of the pre-peritoneal adhesions. Bannenberg et al. observed significant increases in cardiac output during intraperitoneal CO_2_ insufflation in pigs (28); in contrast, moderate decreases in cardiac output during intraperitoneal insufflation have been described in humans (29). However, the prospective effects of prolonged intra- and extraperitoneal CO_2_ insufflation on hemodynamic and gas exchanges have not been evaluated in patients undergoing total RARP, resulting in significant but clinically irrelevant hemodynamic alterations.

We hypothesized that the application of a low CO_2_ pressure could lead to a reduced absorption of the gas into the bloodstream. This could be due to the CO_2_ insufflation pressure being lower than the VCP value (10 mmHg). Our data only showed differences in PaCO_2_ absorption between the two groups at T3 (after DVC manipulation). Therefore, venous absorption of CO_2_ could be proposed as an adjunctive cause of hypercapnia at this surgical step. However, this was not associated with a statistical difference in arterial pH, because the minute ventilation volume was adjusted according to repeated blood gas analyses, without clinically significant changes in the acid-base balance. A recent RCT suggests that TP-RARP is safer than EP-RARP from an anesthesiologic point of view due to a major risk of acidosis in the latter. However, this finding could be due to the non-use of technologies that maintain a constant intra-cavity pressure during the operating phases (9). In fact, the cyclic decrease/increase in intra-cavity pressure produced by a suction device is an adjunctive mechanism of reabsorption (7, 9). This may have a lesser impact when the AirSeal® device is used, as it offers a more stable cavity, reducing episodes of pressure loss compared to the standard port system (11). The effects of the application of the AirSeal® system in abdominal low-pressure laparoscopy have not yet been reported (30). The non-use of this device may represent a major confounding factor when an EP surgery is preferred, especially in cases of intraperitoneal leakage. In order to reduce the impact of this variable, we excluded patients with evident intraperitoneal leakage.

The use of a ventilatory strategy tailored to maintain adequate gas exchange is crucial, especially in the Trendelenburg position, which is commonly employed during RARP to enhance surgical exposure. The steep Trendelenburg position, while beneficial for the surgeon, can reduce functional lung capacity and increase intrathoracic pressure even if the effects on dead space ventilation and venous admixture are small (31, 32), complicating the management of ventilation (20, 33, 34). Maintaining low tidal volumes and applying adequate positive end-expiratory pressure (PEEP) (35) are essential to minimize the risks of barotrauma and volutrauma (36). The EP technique, unlike the TP technique, allows the surgeon to reduce the angle of the Trendelenburg position because the maintenance of gas pressure in the space of Retzius and the integrity of the peritoneum act as a natural retractor, avoiding bowel displacement into the operative field and decreasing the overall complication rate (17, 21, 37). The decrease in lung volume in this position has been described to be approximately 15% (38), while the Tidal volume can be easily increased when using an angle lower than 20°. Unexpectedly, the EP approach has been reported to lead to significantly higher CO_2_ absorption than the TP approach, despite reducing the angle of the Trendelenburg position and the console time (7, 9, 23, 39, 40).

Our perioperative parameters and complication rate are in line with those reported in previous EP and TP series (41, 42). Our results demonstrate that the application of a constant low gas pressure during EP-RARP reduces CO_2_ absorption. The use of the AirSeal® system allows for the maintenance of a stable pressure in the surgical field, avoiding positive and negative pressure peaks. Thus, EP-RARP with constant low pressure is a good surgical strategy for patients with underlying chronic obstructive or restrictive pulmonary disease and obese patients (32, 33, 42).

The strength of our study is that a single surgeon performed the surgeries in the cohort and the same technique was performed for all patients, with the number of confounding factors reduced due to strict inclusion criteria. However, the main limitations of our study were the lack of a control group, consisting of patients who underwent TP-RARP with constant low-pressure CO_₂_ insufflation, and its retrospective design, which may have introduced selection bias. However, the consecutive enrollment of the patients and the comparable baseline characteristics in the two groups partially mitigate this limitation. The study also had a small sample size and was limited to patients with specific biological and disease characteristics.

## 5 Conclusions

This study suggests that EP-RARP can be safely performed using low-pressure CO_2_ insufflation. From the anesthesiologic point of view, using low pressure in RARP reduces CO_2_ absorption during EP surgery without increasing the complication rate. Our data should be interpreted with caution due to the highly selective cohort, but this approach could be highly beneficial in patients with anesthesiologic complications who require RARP.

## Data Availability

The raw data supporting the conclusions of this article will be made available by the authors without undue reservation.

## References

[1] Zhou CK Check DP Lortet-Tieulent J Laversanne M Jemal A Ferlay J Prostate cancer incidence in 43 populations worldwide: an analysis of time trends overall and by age group. Int J Cancer. (2016) 138:1388–400. 10.1002/ijc.29894 26488767 PMC4712103

[2] Bianchi L Gandaglia G Fossati N Larcher A Pultrone C Turri F Oncologic outcomes in prostate cancer patients treated with robot-assisted radical prostatectomy: results from a single institution series with more than 10 years follow up. Minerva Urologica e Nefrologica. (2019):71:38–46. 10.23736/S0393-2249.18.03285-X 30547906

[3] Schuessler WW Schulam PG Clayman RV Kavoussi LR. Laparoscopic radical prostatectomy: initial short-term experience. Urology. (1997) 50:854–7. 10.1016/S0090-4295(97)00543-8 9426713

[4] Paladini A Cochetti G Colau A Mouton M Ciarletti S Felici G The challenges of patient selection for prostate cancer focal therapy: a retrospective observational multicentre study. Current Oncology. (2022) 29:6826–33. 10.3390/curroncol29100538 36290815 PMC9600719

[5] Mearini E Cirocchi R Cochetti G. Robot-assisted surgery in urology: the show must go on. Appl Sci. (2019) 9:844. 10.3390/app9050844

[6] de Vermandois JAR Cochetti G del Zingaro M Santoro A Panciarola M Boni A Evaluation of surgical site infection in mini-invasive urological surgery. Open Med. (2019) 14:711–8. 10.1515/med-2019-0081 PMC6749724 31572804

[7] Meininger D Byhahn C Wolfram M Mierdl S Kessler P Westphal K. Prolonged intraperitoneal versus extraperitoneal insufflation of carbon dioxide in patients undergoing totally endoscopic robot-assisted radical prostatectomy. Surg Endosc. (2004) 18:829–33. 10.1007/s00464-003-9086-9 15216868

[8] Binder J Bräutigam R Jonas D Bentas W. Robotic surgery in urology: fact or fantasy? BJU Int. (2004) 94:1183–7. 10.1046/j.1464-410x.2004.05130.x 15613161

[9] Dal Moro F Crestani A Valotto C Guttilla A Soncin R Mangano A Anesthesiologic effects of transperitoneal versus extraperitoneal approach during robot-assisted radical prostatectomy: results of a prospective randomized study. Int Braz J Urol. (2015) 41:466–72. 10.1590/S1677-5538.IBJU.2014.0199 26200539 PMC4752139

[10] Bongiolatti S Corzani R Borgianni S Meniconi F Cipollini F Gonfiotti A Long-term results after surgical treatment of the dominant lung adenocarcinoma associated with ground-glass opacities. J Thorac Dis. (2018) 10(8):4838–48. 30233857 10.21037/jtd.2018.07.21 PMC6129865

[11] Horstmann M Horton K Kurz M Padevit C John H. Prospective comparison between the AirSeal® system valve-less trocar and a standard Versaport™ plus V2 trocar in robotic-assisted radical prostatectomy. J Endourol. (2013) 27:579–82. 10.1089/end.2012.0632 23186377

[12] Cochetti G Boni A Barillaro F Pohja S Cirocchi R Mearini E. Full neurovascular sparing extraperitoneal robotic radical prostatectomy: our experience with PERUSIA technique. J Endourol. (2017) 31(1):32–7. 27824258 10.1089/end.2016.0477

[13] D’Amico AV. Risk-based management of prostate cancer. N Engl J Med. (2011) 365:169–71. 10.1056/NEJMe1103829 21751910

[14] Charlson ME Pompei P Ales KL MacKenzie CR. A new method of classifying prognostic comorbidity in longitudinal studies: development and validation. J Chronic Dis. (1987) 40:373–83. 10.1016/0021-9681(87)90171-8 3558716

[15] Knuf KM Maani CV Cummings AK. Clinical agreement in the American Society of Anesthesiologists physical status classification. Perioper Med. (2018) 7:14. 10.1186/s13741-018-0094-7 PMC6008948 29946447

[16] Dindo D Demartines N Clavien P-A. Classification of surgical complications. Ann Surg. (2004) 240:205–13. 10.1097/01.sla.0000133083.54934.ae 15273542 PMC1360123

[17] Cochetti G del Zingaro M Ciarletti S Paladini A Felici G Stivalini D New evolution of robotic radical prostatectomy: a single center experience with PERUSIA technique. Appl Sci. (2021) 11:1513. 10.3390/app11041513

[18] Hinev AI Anakievski D Kolev NH Hadjiev VI. Validation of nomograms predicting lymph node involvement in patients with prostate cancer undergoing extended pelvic lymph node dissection. Urol Int. (2014) 92:300–5. 10.1159/000354323 24480972

[19] Schiavina R Chessa F Borghesi M Gaudiano C Bianchi L Corcioni B State-of-the-art imaging techniques in the management of preoperative staging and re-staging of prostate cancer. Int J Urol. (2019) 26:18–30. 10.1111/iju.13797 30238516

[20] Nunn JF Hill DW. Respiratory dead space and arterial to end-tidal CO_2_ tension difference in anesthetized man. J Appl Physiol. (1960) 15:383–9. 10.1152/jappl.1960.15.3.383 14427915

[21] Balasubramanian S Shiang A Vetter JM Henning GM Figenshau RS Kim EH. Comparison of three approaches to single-port robot-assisted radical prostatectomy: our institution’s initial experience. J Endourol. (2022) 36:1551–8. 10.1089/end.2022.0330 36017625 PMC9718430

[22] Gandaglia G Karl A Novara G de Groote R Buchner A D’Hondt F Perioperative and oncologic outcomes of robot-assisted vs. Open radical cystectomy in bladder cancer patients: a comparison of two high-volume referral centers. European Journal of Surgical Oncology (EJSO). (2016) 42:1736–43. 10.1016/j.ejso.2016.02.254 27032295

[23] Glascock JM Winfield HN Lund GO Donovan JF Ping STS Griffiths DL. Carbon dioxide homeostasis during transperitoneal or extraperitoneal laparoscopic pelvic lymphadenectomy: a real-time intraoperative comparison. J Endourol. (1996) 10:319–23. 10.1089/end.1996.10.319 8872727

[24] Neupane S Bray F Auvinen A. National economic and development indicators and international variation in prostate cancer incidence and mortality: an ecological analysis. World J Urol. (2017) 35:851–8. 10.1007/s00345-016-1953-9 27744614

[25] di Trapani E Sanchez-Salas R Gandaglia G Rocchini L Moschini M Lizee D A nomogram predicting the cancer-specific mortality in patients eligible for radical cystectomy evaluating clinical data and neoadjuvant cisplatinum-based chemotherapy. World J Urol. (2016) 34:207–13. 10.1007/s00345-015-1640-2 26198750

[26] Kanwer DB Kaman L Nedounsejiane M Medhi B Verma GR Bala I. Comparative study of low pressure versus standard pressure pneumoperitoneum in laparoscopic cholecystectomy – a randomised controlled trial. Trop Gastroenterol. (2009) 30:171–4. 20306755

[27] Moschini M Gandaglia G Dell’Oglio P Fossati N Cucchiara V Burgio G Incidence and predictors of 30-day readmission in patients treated with radical cystectomy: a single center European experience. Clin Genitourin Cancer. (2016) 14:e341–6. 10.1016/j.clgc.2015.12.017 26797584

[28] Bannenberg JJG Rademaker BMP Froeling FMJA Meijer DW. Hemodynamics during laparoscopic extra- and intraperitoneal insufflation. Surg Endosc. (1997) 11:911–4. 10.1007/s004649900485 9294271

[29] Montorsi F Gandaglia G Chapple C Cruz F Desgrandchamps F Llorente C. Effectiveness and safety of silodosin in the treatment of lower urinary tract symptoms in patients with benign prostatic hyperplasia: a European phase IV clinical study (SiRE study). Int J Urol. (2016) 23:572–9. 10.1111/iju.13088 26969887

[30] Sroussi J Elies A Rigouzzo A Louvet N Mezzadri M Fazel A Low pressure gynecological laparoscopy (7 mmHg) with AirSeal® system versus a standard insufflation (15 mmHg): a pilot study in 60 patients. J Gynecol Obstet Hum Reprod. (2017) 46:155–8. 10.1016/j.jogoh.2016.09.003 28403972

[31] Park E Koo B Min K Nam S. The effect of pneumoperitoneum in the steep Trendelenburg position on cerebral oxygenation. Acta Anaesthesiol Scand. (2009) 53:895–9. 10.1111/j.1399-6576.2009.01991.x 19426238

[32] Awad H Santilli S Ohr M Roth A Yan W Fernandez S The effects of steep Trendelenburg positioning on intraocular pressure during robotic radical prostatectomy. Anesth Analg. (2009) 109:473–8. 10.1213/ane.0b013e3181a9098f 19608821

[33] Capogrosso P Ventimiglia E Serino A Stabile A Boeri L Gandaglia G Orgasmic dysfunction after robot-assisted versus open radical prostatectomy. Eur Urol. (2016) 70:223–6. 10.1016/j.eururo.2015.10.046 26572706

[34] Nunn J. Prediction of carbon dioxide tension during anaesthesia. Anaesthesia. (1960) 15:123–33. 10.1111/j.1365-2044.1960.tb13317.x 14427918

[35] Cammarota G Simonte R De Robertis E. PEEP-induced alveolar recruitment in patients with COVID-19 pneumonia: take the right time! Crit Care. (2021) 25:163. 10.1186/s13054-021-03573-x 33931092 PMC8086221

[36] Longhini F Simonte R Vaschetto R Navalesi P Cammarota G. Reverse triggered breath during pressure support ventilation and neurally adjusted ventilatory assist at increasing propofol infusion. J Clin Med. (2023) 12:4857. 10.3390/jcm12144857 37510970 PMC10381884

[37] Paladini A Cochetti G Felici G Russo M Saqer E Cari L Complications of extraperitoneal robot-assisted radical prostatectomy in high-risk prostate cancer: a single high-volume center experience. Front Surg. (2023) 10:1157528. 37066016 10.3389/fsurg.2023.1157528 PMC10098012

[38] Case EH Stiles JA. The effect of various surgical positions on vital capacity. Anesthesiology. (1946) 7:29–31. 10.1097/00000542-194601000-00005 21013143

[39] Atug F Castle EP Woods M Srivastav SK Thomas R Davis R. Transperitoneal versus extraperitoneal robotic-assisted radical prostatectomy: is one better than the other? Urology. (2006) 68:1077–81. 10.1016/j.urology.2006.07.008 17095060

[40] Davis JW Achim M Munsell M Matin S. Effectiveness of postgraduate training for learning extraperitoneal access for robot-assisted radical prostatectomy. J Endourol. (2011) 25:1363–9. 10.1089/end.2011.0052 21745117 PMC3180764

[41] Xylinas E Durand X Ploussard G Campeggi A Allory Y Vordos D Evaluation of combined oncologic and functional outcomes after robotic-assisted laparoscopic extraperitoneal radical prostatectomy: trifecta rate of achieving continence, potency and cancer control. Urol Oncol: Semin Orig Invest. (2013) 31:99–103. 10.1016/j.urolonc.2010.10.012 21719321

[42] Ficarra V Novara G Artibani W Cestari A Galfano A Graefen M Retropubic, laparoscopic, and robot-assisted radical prostatectomy: a systematic review and cumulative analysis of comparative studies. Eur Urol. (2009) 55:1037–63. 10.1016/j.eururo.2009.01.036 19185977

